# Individual- and area-level characteristics associated with alcohol-related mortality among adult Lithuanian males: A multilevel analysis based on census-linked data

**DOI:** 10.1371/journal.pone.0181622

**Published:** 2017-07-21

**Authors:** Pavel Grigoriev, Domantas Jasilionis, Daumantas Stumbrys, Vladislava Stankūnienė, Vladimir M. Shkolnikov

**Affiliations:** 1 Max Planck Institute for Demographic Research (Rostock, Germany); 2 Demographic Research Centre, Vytautas Magnus University (Kaunas, Lithuania); 3 National Research University Higher School of Economics (Moscow, Russian Federation); University of Toronto, CANADA

## Abstract

**Background:**

Although excessive alcohol-related mortality in the post-Soviet countries remains the major public health threat, determinants of this phenomenon are still poorly understood.

**Aims:**

We assess simultaneously individual- and area-level factors associated with an elevated risk of alcohol-related mortality among Lithuanian males aged 30–64.

**Methods:**

Our analysis is based on a census-linked dataset containing information on individual- and area-level characteristics and death events which occurred between March 1^st^, 2011 and December 31^st^, 2013. We limit the analysis to a few causes of death which are directly linked to excessive alcohol consumption: accidental poisonings by alcohol (X45) and liver cirrhosis (K70 and K74). Multilevel Poisson regression models with random intercepts are applied to estimate mortality rate ratios (MRR).

**Results:**

The selected individual-level characteristics are important predictors of alcohol-related mortality, whereas area-level variables show much less pronounced or insignificant effects. Compared to married men, never married (MRR = 1.9, CI:1.6–2.2), divorced (MRR = 2.6, CI:2.3–2.9), and widowed (MRR = 2.4, CI: 1.8–3.1) men are disadvantaged groups. Men who have the lowest level of educational attainment have the highest mortality risk (MRR = 1.7 CI:1.4–2.1). Being unemployed is associated with a five-fold risk of alcohol-related death (MRR = 5.1, CI: 4.4–5.9), even after adjusting for all other individual variables. Lithuanian males have an advantage over Russian (MRR = 1.3, CI:1.1–1.6) and Polish (MRR = 1.8, CI: 1.5–2.2) males. After adjusting for all individual characteristics, only two out of seven area-level variables—i.e., the share of ethnic minorities in the population and the election turnout—have statistically significant direct associations. These variables contribute to a higher risk of alcohol-related mortality at the individual level.

**Conclusions:**

The huge and increasing socio-economic disparities in alcohol-related mortality indicate that recently implemented anti-alcohol measures in Lithuania should be reinforced by specific measures targeting the most disadvantaged population groups and geographical areas.

## Introduction

Alcohol is known to be one the main contributors to the mortality gap between eastern and western Europe, as well as a major public health threat in the post-Soviet countries. [1–5] The recent changes in alcohol consumption patterns in eastern Europe appear to be the result of interactions between shifts in individual behaviour and the effects of alcohol policies. These patterns have long been reliable predictors of premature mortality across the region, including in Lithuania. [6–9]

Male premature mortality rose sharply during the socio-economic crisis of the early 1990s which accompanied Lithuania’s transition from a socialist to a market economy, but improved markedly during the second half of the 1990s. Despite the generally positive socio-economic trends and promising political changes in the period that followed—such as Lithuania’s entry into the European Union (EU) in 2004—male mortality continued to rise during 2000–2007. [10] The liberalisation of alcohol control policies led to enormous growth in alcohol consumption, and, consequently, to increases in both alcohol-related and overall male mortality. [10,11] These trends were not reversed until 2008, following the implementation of a number of anti-alcohol measures. [9,12,13] While alcohol-related and overall adult mortality has declined substantially in recent years, the problem of excessive alcohol consumption in Lithuania continues to be acute. According to a report of the World Health Organization [14], Lithuania is ranked third in the world in terms of total alcohol consumption (15.4 l per capita), after Moldova (16.8 l) and neighbouring Belarus (17.5 l). Within the European Union, Lithuania not only has the highest level of alcohol consumption, but also the highest rate of alcohol-related mortality. [15] Reliable population-level evidence regarding the socio-demographic and socio-economic determinants of alcohol-related mortality in Lithuania is scarce, and the few studies that exist were based on data from the 1990s or the beginning of the 2000s. [16–18] Relative to population-level studies, survey-based studies have significant disadvantages: for example, they tend to rely on self-reported information on alcohol consumption, and often exclude the most vulnerable population groups.

In this study, we aim to assess both the individual- and the area-level (contextual) factors associated with an elevated risk of alcohol-related mortality among males of working ages in Lithuania during 2011–2013. To our knowledge, this is the first study on this topic in the post-Soviet region which employs the multilevel approach and makes use of a unique census-linked dataset. The large body of evidence obtained from this research is mostly based on either national-level data [1,6,7,19,20] or small-scale epidemiological studies which focused primarily on Russia. [3,4,21] Importantly, we seek to provide new insights into the determinants of alcohol-related mortality shortly after the implementation of anti-alcohol policies in 2008–2009 which also coincided with the severe economic crisis of 2008–2009. Using a multilevel approach provides new analytical opportunities such as simultaneous modelling of area-level (contextual) and individual effects ensuring more robust estimations of explanatory variables. [22] There is consistent evidence of modest neighbourhood effects on health after adjusting for individual socio-economic status. [23] A recent study by Grigoriev and colleagues [24] has suggested that there is a notable geographical clustering of alcohol-related mortality across Lithuanian municipalities. The chosen multilevel modelling approach extends prior evidence by a) identifying possible determinants of the variation of alcohol-related mortality across municipalities and b) exploring whether municipality-level effects remain significant after controlling for individual-level characteristics. These new findings provide new insights on determinants and possible mechanisms behind excessive adult male mortality due to alcohol-related causes of death in Lithuania and other post-soviet countries.

## Data and methods

### Census-linked data

This study is based on an aggregated census-linked mortality dataset provided by Statistics Lithuania. The linkages between the individual 2011 census death and emigration records between March 1^st^, 2011 (2011 Census) and December 31^st^, 2013 were implemented by employees of Statistics Lithuania, who have permission to work with confidential individual data. The linkage allowed to establish the census-based socio-demographic and socio-economic status for each individual who died or emigrated during the observation period. The dates of death and emigration were used to estimate exact numbers of person-years of exposure to risk of dying due to alcohol-related causes of death.

For the purposes of this study, Statistics Lithuania provided a census-linked dataset in the format of frequencies which refer to aggregated numbers of deaths and person years of exposure to risk across all possible combinations of available socio-demographic and socio-economic variables (including municipality variables).

### Definition of alcohol-related death

Prior international assessment of country-specific completeness and reliability of statistics on causes of death has indicated that such data in Lithuania are complete and accurate. [25] However, in order to avoid potential distortions due to specifics of registration and possible misclassification of causes of death [26,27], we deal only with few well-defined causes of death which are known to be either fully attributable or causally linked to alcohol [28]. These causes include alcoholic and other cirrhosis of the liver (items K70 and K74 of the ICD–10) and accidental poisonings by alcohol (X45). The selected causes are known to be sensitive indicators of alcohol consumption, and they are also strongly correlated to overall mortality in the region. [6,7]

### Individual and area-level variables

The census-based individual variables include *age* (control variable), *place of residence* (urban, rural), *marital status* (married, never married, divorced, and widowed), *education* (higher, secondary, and lower than secondary), *employment status* (employed, unemployed, inactive/disabled, other inactive, and unknown), and *nationality* (Lithuanian, Russian, Polish, and other).

Area-level variables for 60 Lithuanian municipalities are either obtained from official statistical publications [29] or calculated directly from 2011 census data. The contextual characteristics have been selected to cover four major domains: socio-economic conditions and deprivation, family cohesion, cultural context, and civic participation. Based on previous research [30] these domains are expected to be associated with alcohol-related mortality. The variables of the first domain (socioeconomic conditions and deprivation) include *unemployment rate*, *average salary ratio* (ratio of annual net-salary to national average), *share of manual workers*, and *share of individuals receiving social benefits*. The variable *share of single households* (proportion of households having only one person) was selected to reflect the degree of family cohesion, while the variable *share of non-Lithuanian population* (proportion of other ethnicities in the total population) is used as a proxy of sociocultural context. Finally, *election turnout* (participation rate in municipal elections) is used to measure civic participation. Each of the contextual variables was categorised into tertiles (low, medium, and high), with each group containing roughly 20 observations (municipalities).

### Statistical analyses

The final dataset used in the analysis contains data on 1424 death events and 1.869 million person-years of exposure. We restrict our analysis to males aged 30–64. The individual characteristics (except the date of death) stem from the census, and remain fixed at the census baseline. The lower age limit was chosen based on the assumption that changes in socioeconomic status (especially in education) are negligible after age 30.

Two-level models are fitted using multilevel Poisson regression with a log link and logarithm of person-years set as an offset. Poisson regression is a convenient approach for estimating relative rate ratios using aggregated count data (deaths and person years of exposure split by socio-demographic and socio-economic variables). Similar methodology has been widely used in numerous well-known international and national studies addressing relative socio-economic inequalities in Europe and elsewhere [31–33]. In our study we apply a Poisson regression multilevel model with random intercepts, assuming that the intercept is allowed to vary across municipalities. For the detailed specification of the model, see [22]. Multilevel models are fitted using R package *lme4*, function *glmer*. [34] The results appear in the form of mortality rate ratios (MRR) which are exponentiated *β* coefficients.

As the starting point of our analysis we ran an empty model, and calculated the intra-class correlation (ICC). [22] The estimated value of ICC of 0.0964 suggests that about 10 per cent of inter-individual variation in the risk of alcohol-related death is attributable to living in a certain municipality. The remaining variation is explained by individual characteristics (observed and unobserved).

We first fitted the model containing only individual-level variables (including age) to assess their influence on the risk of alcohol-related death. We then assessed the impact of each of the area-level variables individually, without simultaneously controlling for other available contextual characteristics. Here, we fitted two types of models: 1) a contextual variable plus age (age-adjusted model), and 2) a contextual variable plus age and all individual variables (fully adjusted model). This strategy allowed us to assess 1) the individual effects, 2) the unadjusted contextual effects (ecological effects without controlling for the individuals’ characteristics, except age), and, finally, 3) the fully adjusted contextual effects (after controlling for all of the individual variables, including age).

To show the potential for the elimination of mortality inequalities in a hypothetical scenario in which members of all socio-economic groups had the same mortality as their counterparts in the highest education and economic status groups, population attributable fractions (PAFs) are calculated using a conventional formula. [35]

## Results

The main results of our multilevel analysis of individual- and area-level variables are summarized in Tables 1 and 2, respectively. The supplementary results for each of the 15 models presented in Tables 1 and 2 appear in S1 Appendix. Model 1 shown in S1 Appendix corresponds to the model presented in Table 1. Models 2–8 and Models 9–15 correspond to age-adjusted (Model A) and fully-adjusted (Model B) models presented in Table 2.

**Table 1:** Person-years of exposure, number of death and mortality rate ratios (MRR) of alcohol-related mortality; Lithuanian males aged 30–64, 2011–2013 (all individual characteristics).

	Person-years of exposure (x1000)	Deaths	MRR	95% confidence limits	
				lower	upper
**Place of residence**					
Urban	1221	880	1.000	-	-
Rural	648	544	0.796***	0.696	0.911
**Marital status**					
Married	1291	594	1.000	-	-
Never married	293	312	1.878***	1.617	2.181
Divorced	255	455	2.596***	2.291	2.941
Widowed	30	63	2.384***	1.834	3.100
**Education**					
Higher	418	132	1.000	-	-
Secondary	1176	960	1.394***	1.154	1.683
Lower than secondary	275	332	1.728***	1.393	2.143
**Economic activity**					
Employed	1235	287	1.000	-	-
Unemployed	360	563	5.074***	4.376	5.884
Inactive, disabled	123	321	7.540***	6.366	8.932
Other inactive	141	239	5.311***	4.399	6.412
Unknown	10	14	5.120***	2.934	8.933
**Nationality**					
Lithuanian	1551	1026	1.000	-	-
Russian	126	136	1.287***	1.064	1.557
Polish	129	212	1.819***	1.491	2.221
Other	63	50	1.077	0.802	1.446

Source: own calculations; all variables were entered in the model simultaneously and controlled for age

p<0.01 ***

p<0.05 **

p<0.10 *

**Table 2:** Person-years of exposure, number of death, and mortality rate ratios (MRR) of alcohol-related mortality; Lithuanian males aged 30–64, 2011–2013 (area-level characteristics).

	Person-years of exposure (x1000)	Deaths	Model A (age adjusted)			Model B (adjusted for age and all individual characteristics)		
			MRR	95% confidence limits		MRR	95% confidence limits	
				lower	upper		lower	upper
**Share of non-Lithuanian population, per cent**								
Low (0.8–2.2]	383	251	1.000	-	-	1.000	-	-
Medium (2.2–5.7]	532	374	0.987	0.770	1.265	1.040	0.851	1.272
High (5.7–89.2]	954	799	1.393***	1.093	1.775	1.371***	1.120	1.677
**Share of single households, per cent**								
Low (23.2–30.2]	552	440	1.000	-	-	1.000	-	-
Medium (30.2–32.9]	469	304	0.919	0.709	1.191	0.888	0.723	1.091
High (32.9–37.7]	848	680	1.087	0.846	1.396	1.086	0.895	1.318
**Share of individuals receiving social benefits, per cent**								
Low (3.1–7.6]	1015	715	1.000	-	-	1.000	-	-
Medium (7.6–8.4]	452	331	1.041	0.812	1.335	0.972	0.794	1.191
High (8.4–11.9]	402	378	1.247*	0.971	1.601	1.066	0.867	1.311
**Unemployment rate, per cent**								
Low (6.7–12.6]	1010	723	1.000	-	-	1.000	-	-
Medium (12.6–15.0]	429	312	0.972	0.753	1.255	0.876	0.715	1.074
High (15.0–20.0]	430	389	1.142	0.887	1.469	0.972	0.794	1.189
**Election turnout, per cent**								
Low (33.5–43.5]	918	564	1.000	-	-	1.000	-	-
Medium (43.5–48.6]	627	512	1.347***	1.096	1.654	1.233**	1.033	1.470
High (48.6–66.6]	324	348	1.646***	1.330	2.037	1.357***	1.121	1.643
**Average salary ratio**								
Low (0.70–0.80]	366	295	1.000	-	-	1.000	-	-
Medium (0.80–0.86]	419	365	1.006	0.778	1.301	1.021	0.830	1.255
High (0.86–1.18]	1084	764	0.979	0.752	1.275	1.106	0.892	1.371
**Share of manual workers, per cent**								
Low (6.1–10]	1150	793	1.000	-	-	1.000	-	-
Medium (10–11.2]	417	363	1.157	0.909	1.473	1.056	0.867	1.286
High (11.2–14.8]	302	268	1.232	0.957	1.587	1.148	0.931	1.417

Source: own calculations; Each of the contextual variables was entered in the model separately; All area-level variables refer to the year 2011. The initial data were obtained from Statistics Lithuania [28], except the variable *share of non-Lithuanian population*, which was calculated directly from 2011 census data.

p<0.01 ***

p<0.05 **

p<0.10 *

Table 1 demonstrates the importance of the selected individual-level socio-demographic and socio-economic characteristics. In overall, after controlling for the individual characteristics mortality variation across municipalities reduced by more than a half (55.1 per cent) as compared to the empty model (see S1 Appendix). We found that never married, divorced, and widowed men have a much higher risk (1.9–2.6 times) of alcohol-related mortality than married men. Compared to the highest education group, the least educated males also showed substantial (1.8 times) excess mortality risk. Nationality was also an important predictor of alcohol-related mortality in Lithuania. Even after controlling for major compositional characteristics such as education, the mortality risk was almost twice as high among Poles (MRR = 1.8, CI [1.5–2.2]) as among ethnic Lithuanians. Russians displayed a less pronounced disadvantage relative to the native population (MRR = 1.3, CI [1.1–1.6]).

However, the most striking differentials based on individual-level effects were observed for economic activity status. Being unemployed or otherwise economically inactive, or having an unknown economic activity status increased the risk of alcohol-related death by a factor of five. Our finding that disabled economically inactive males were seven times more likely to die from alcohol-related causes than employed males was even more unexpected. It is also interesting to note that there was no advantage of urban males over their rural counterparts. On the opposite, when the socioeconomic factors were accounted for, Lithuanian rural males were actually less likely to die from the alcohol-related causes (MRR = 0.8, CI [0.7–0.9].

**Fig 1:**
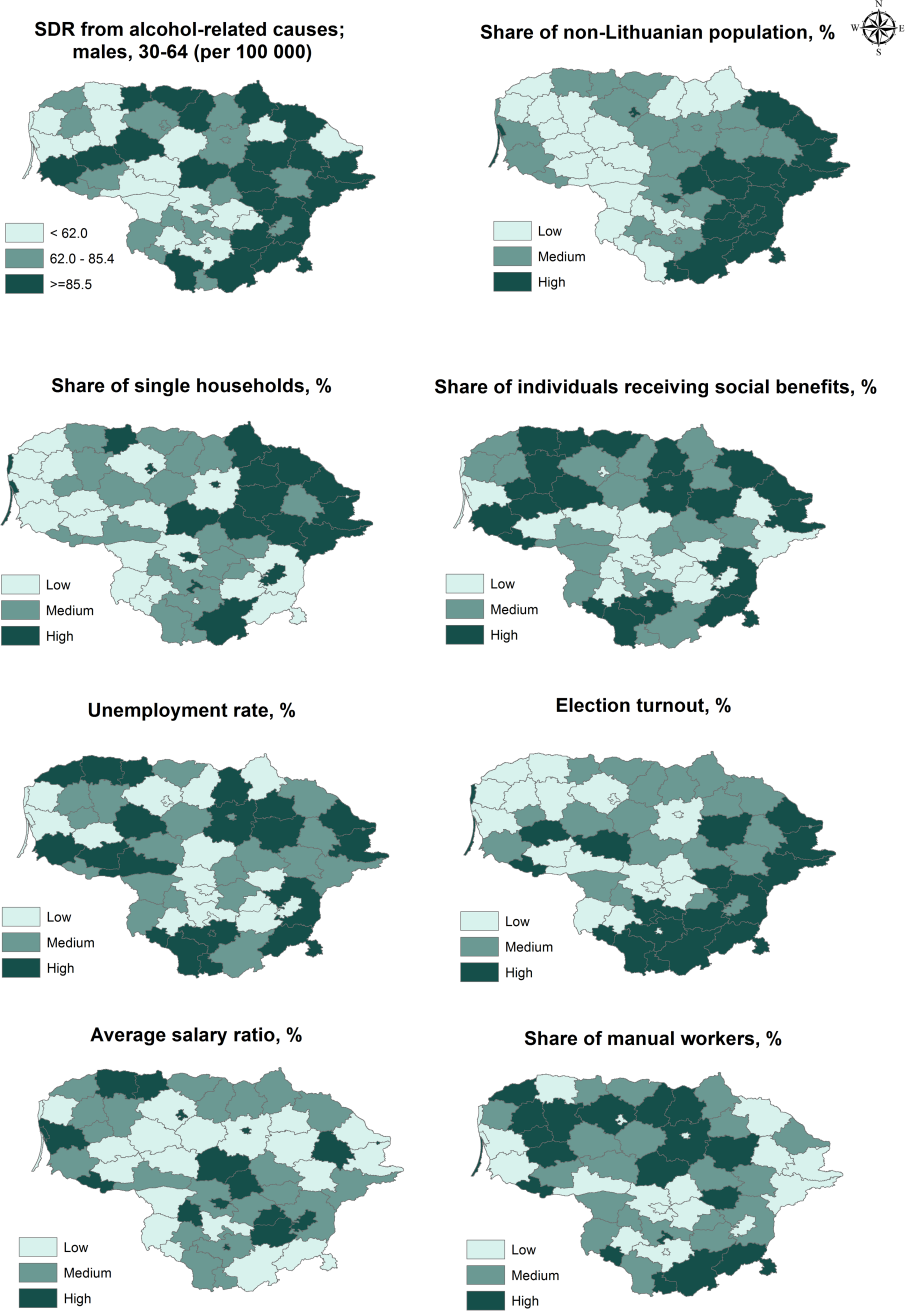
Alcohol-related male mortality rates and area-level characteristics across Lithuanian municipalities. Sources: *SDR from alcohol-related causes* and *share of non-Lithuanian population were calculated from 2011 census data; remaining characteristics–*Statistics Lithuania [29].

Fig 1 depicts the geographical distribution of alcohol-related mortality and area-level variables, and demonstrates clear evidence of spatial dependence. The mortality rates tend to be higher in eastern and south-eastern Lithuania. These regions also have relatively high proportions of the population who are non-Lithuanian, and high levels of turnout for municipal elections. The proportion of households led by singles is clearly higher in eastern Lithuania and in the major Lithuanian cities than in other parts of the country (see the S2 Appendix for the detailed country administrative divisions). The Lithuanian cities are also situated in the top quintile for *average salary ratio*. There is no notable clustering in the geographical distributions of the other contextual variables.

As it can be inferred from S1 Appendix the inclusion of some area-level variables significantly improves the explanatory power of the model. For example, when variable *election turnout* controlled for age was introduced in the model variance of random intercepts reduced by 44 per cent (Model 7). Further controlling for the individual characteristics reduced variance by 72 per cent (Model 14).

Table 2 shows both the unadjusted (Model A) and adjusted (Model B) effects of the contextual characteristics on the risk of alcohol-related death.

As for Model A, the risk of alcohol-related death was higher and statistically significant in municipalities with a relatively large share (more than six per cent) of ethnic minorities in the population, with a comparatively large share of the population receiving social benefits, and with a relatively high election turnout.

After controlling for all of the individual characteristics (Model B) the effects of the *share of non-Lithuanian nationality* and *election turnout* remained statistically significant, while the effect of the share of people receiving social benefits was no longer statistically significant.

## Discussion

### Main results

To our knowledge, this is a first population-level study providing reliable evidence on both individual- and area-level determinants of alcohol-related mortality in a post-soviet country. The study is based on high quality census-linked data covering the entire male population in Lithuania which include reliable and representative information on the socio-economic status of the deceased. The census-linked approach allowed to avoid numerous problems related to the quality of socio-demographic and socio-economic information provided on death records which often lead to the numerator-denominator bias. [31,36–38].

Our study highlights the importance for mortality of certain individual-level characteristics, such as economic activity status, marital status, and education. Our findings also show, however, that after adjusting for individual-level variables, most of the area-level characteristics have a relatively small impact. At the individual level, the biggest relative mortality rate ratios were found for the following categories of men: those who are unemployed or otherwise inactive; those with a missing economic status; and, in particular, those who are disabled and inactive. Several studies suggested that the relationship between unemployment and mortality, alcohol may act as an intermediate factor; a “stress reliever” that helps men to cope with challenging situations such as the loss of a job. In addition, the possibility of reverse causality (e.g., that individuals who are heavy drinkers are more likely than others to become unemployed or disabled) and of effects of unmeasured confounders cannot be excluded. [39–41] Studies on working-age men in Russia described a downward trajectory ending in death often begins with the loss of a job and the accompanying decline in the social position. [42] But the trajectory may also start with a pattern of alcohol abuse, which in time leads to even more alcohol consumption and a deterioration in the individual’s socio-economic, family, and health situations.

A comparison of the observed results from this study with the corresponding estimates for 2001–2005 suggests that mortality differences by economic activity status increased dramatically: from 3.1 to 4.1 times for lower educated males, from 3.5 to 6.7 times for unemployed males, from 2.8 to 6.5 times for other inactive males, and from 3.6 to 10 times for inactive disabled males. [18,43] It is especially striking that the substantial growth in excess alcohol-related mortality among the unemployed and other inactive males is shown to have occurred immediately after the implementation of anti-alcohol measures in 2007–2009. Our results regarding the effects of education are in line with those of previous research. Men with the lowest level of educational attainment are shown to be the most disadvantaged group. Similar effects of education on mortality have been consistently established for both western and eastern Europe, but they appear to be more pronounced in the post-communist countries. [32] There are several major hypotheses which seek to explain how education influences health-related behaviours (including alcohol consumption) and subsequent mortality risk. Mirowsky and Ross [44–46] highlighted the importance of individuals acquiring particular skills in the early stages of life which lead them to engage in more rational health-related behaviours and to gain greater a degree of control over events at later stages of life. Thus, improvements in education could lead to further reductions in alcohol-related harm in Lithuania.

Our analysis also confirms prior findings from various countries showing that men who are never married, divorced or widowed are disadvantaged relative to married men. There is consistent empirical evidence of the protective effect of marriage-related social networks on adult male mortality. [47,48] However, this association is far from being straightforward, as belonging to a certain marital status group (e.g., divorced) may itself be a consequence of drinking (i.e., a health selection effect). [49]

We also find substantial variation in the alcohol-related mortality risk among ethnic groups. Compared to Lithuanians, Polish men are the most deprived group, while excess mortality is much less pronounced among Russian males. Importantly, these differences cannot be explained by compositional differences in education, economic activity status, or an urban versus a rural place of residence. Our results are consistent with those of prior studies which reported varying mortality rates between different ethnic groups in Lithuania. [17,18] Our findings are also in agreement with those of the recent FINBALT Health Monitor survey (2012), which showed that the proportion of men who consume six or more portions of alcohol on a single occasion at least once a week is higher among non-Lithuanians, especially at ages 55–64. [50]

Assessing the area-level effects on mortality risk from alcohol was the other objective of our analysis. Of the seven area-level (contextual) variables which were analysed separately, only two reveal statistically significant results (after including all variables into the model). First, we find that residing in an area with a large share of ethnic minorities in the population increases the risk of dying from alcohol-related causes. This effect can be explained by the specifics of the socio-economic conditions and the socio-cultural environments in these areas, which may be related to the behavioural patterns of certain ethnic minorities. It has also been shown that areas with elevated alcohol-related mortality cross the national border and continue in the neighbouring areas of Belarus. [24] Specific patterns of alcohol consumption related to illegal production and cross-border smuggling of alcohol may explain the worse conditions in these areas (which also have a higher share of ethnic minorities in the population). It is particularly striking that this (in many respects) disadvantaged region surrounds the capital city of Vilnius, which has the best socio-economic indicators in the entire country.

In terms of the effects of civic participation on mortality risk from alcohol, our results contradict those of previous research for other countries. Studies based on Finnish census-linked data have shown that low voter turnout is associated with increased alcohol-related suicide. [51] Our findings indicate that higher election turnout in municipalities is associated with the higher risk of dying from alcohol-related causes of death. This can be explained by specific regional patterns of voting in the south-eastern region (also showing higher alcohol-related mortality), which is dominated by ethnic minority parties benefiting from a very disciplined and loyal electorate. [52,53] Already for a couple of decades, the ethnic and populist parties have been very successful in mobilizing their voters and ensuring the highest election turnout rates in this region despite persisting socio-economic disadvantage and demographic decline. [54] The highest election turnout rates in this socially and economically deprived area also suffering from the highest alcohol-related mortality rates can hardly be interpreted as a reliable measure reflecting real civic participation. Thus, election turnout seems to be a very vague indicator in the Lithuanian context and should be interpreted with great caution.

The results of this study highlight the potential for saving lives through the elimination or reduction in the huge socio-economic inequalities in alcohol-related mortality, and especially in the relative disparities in education and economic activity status. In hypothetical scenarios in which the lower socio-economic groups had the same mortality as their counterparts in the highest education and economic activity status (employed) groups, 56 and 68 per cent of alcohol-related deaths, respectively, would have been avoided.

Anti-alcohol policies in Lithuania must consider both individual- and area-level determinants, and propose specific measures for the most disadvantaged population groups and areas. The study also points to a complex nature of determinants of alcohol-related mortality in Lithuania which requires a wider scale inter-sectorial policies covering health, social, economic, and legal domains.

### Limitations of the study

Our study has several potential limitations which should be acknowledged. First, the population-level census-linked data provide us with only a few (albeit the most important) individual-level variables. First, the census does not include any information on drinking habits or other behavioural or psychosocial characteristics potentially related to the risk of excessive alcohol consumption. These and other unobserved variables may act as either important predictors of mortality risk and/or as confounders which influence the observed effects of the available individual- and area-level variables. However, even detailed survey-based self-reported information on drinking habits and related predictors is often problematic and suffers from various reporting biases. Because of this shortage of explanatory variables, a significant proportion of mortality risk variation (across municipalities) remains unexplained.

Second, it should be noted that the available individual-level characteristics (except for age) are time constant (i.e., fixed at the time of the census). We can assume that at least some of these characteristics (e.g., employment status) will have changed during the period of observation.

Third, we use a very restrictive definition of alcohol-related mortality which excludes several causally linked to alcohol causes of death, such as ischaemic heart disease, external causes, and cancers of the upper aero-digestive tract and liver. [28, 55] Thus, we rely on a conservative estimate of alcohol-related mortality. For example, studies indicate that more than 50 per cent of the deceased from external causes of death were intoxicated by alcohol. [56] Excluding a number of causes causally linked to alcohol [28] explains why we found such steep socioeconomic mortality gradient. It has been shown that this gradient is indeed steeper in alcohol-attributable as compared to all-cause mortality. [57]. Nevertheless, we believe that using such restrictive definition has helped us to avoid some of the problems related to coding and misclassification which were reported by prior studies in Lithuania. [26,27] However, it is also possible that a part of deaths attributable to alcohol was still undercounted. Prior autopsy-based studies conducted in 1980s found that some part of these causes of death such as poisoning by alcohol and chronic alcoholism were reported as ischaemic heart disease and that this may be related to stigmatization of alcohol-related deaths [58].Unfortunately, such evidence or verification studies do not cover more recent periods. There is no reliable data suggesting that possible misclassification may substantially differ across socio-demographic and socio-economic groups. Taking into account that the percentage of autopsies remain high and that we report only relative differences, possible inaccuracies in cause of death should not have any substantial influence on the results.

Finally, although we believe that the modelling strategy used in this study is adequate, alternative approaches (e.g. Bayesian Poisson spatial models) accounting for the dependence of observations based on local proximity in addition to clustering with municipalities could be considered [59].

## Supporting information

S1 Appendix Supplementary information for the models presented in Tables 1 and 2 of the main text. (DOCX)

S2 Appendix Administrative division of Lithuania. (DOCX)
