# Supplementary material for: Individual- and area-level characteristics associated with alcohol-related mortality among adult Lithuanian males: A multilevel analysis based on census-linked data
Source: PLoS One. 2017 Jul 21;12(7):e0181622. doi: 10.1371/journal.pone.0181622 (PMC5521820; doi:10.1371/journal.pone.0181622)
Supplement: S1 Appendix — (DOCX) [file pone.0181622.s001.docx]

**S1 Appendix**

**Supplementary information for the models presented in Tables 1 and 2 of the main text**

| Model | Name | Description | Fixed effects (Intercept) | Variance of random  intercepts | Reduction  in variance (relative to empty model), per cent | Deviance (likelihood ratio test) |
| --- | --- | --- | --- | --- | --- | --- |
| 0 | Empty | *Empty model* | -7.2144 | 0.1067 | - | 11067.9 |
| 1 | Individual | *All individual variables controlled for age (Table 1)* | -10.1737 | 0.0479 | 55.1 | 9279.2 |
| 3 | NONLTU | *Selected area-level variable controlled for age  (Model 1 of Table 2)* | -8.7257 | 0.0863 | 19.1 | 10800.8 |
| 4 | ALONE |  | -8.5812 | 0.0983 | 7.9 | 10806.0 |
| 5 | SOCBEN |  | -8.6617 | 0.0953 | 10.7 | 10805.6 |
| 6 | UNEMPLOY |  | -8.6143 | 0.1010 | 5.3 | 10807.1 |
| 7 | ELECTION |  | -8.7622 | 0.0598 | 44.0 | 10795.4 |
| 8 | RSALARY |  | -8.5765 | 0.1062 | 0.5 | 10808.8 |
| 9 | MANUALW |  | -8.6813 | 0.0951 | 10.9 | 10806.1 |
| 10 | (1) + NONLTU | *Selected area-level variable controlled for age and all individual characteristics (Model 2 of Table 2)* | -10.3581 | 0.0318 | 70.2 | 9269.3 |
| 11 | (1)+ ALONE |  | -10.1737 | 0.0350 | 67.2 | 9274.2 |
| 12 | (1) + SOCBEN |  | -10.1777 | 0.0458 | 57.0 | 9278.4 |
| 13 | (1) + UNEMPLOY |  | -10.1268 | 0.0452 | 57.6 | 9277.4 |
| 14 | (1) + ELECTION |  | -10.2630 | 0.0301 | 71.8 | 9271.7 |
| 15 | (1) + RSALARY |  | -10.2284 | 0.0467 | 56.2 | 9278.2 |
| 16 | (1) + MANUALW |  | -10.2113 | 0.0442 | 58.6 | 9277.6 |

Note: NONLTU- Share of non-Lithuanian population, ALONE - Share of single households, SOCBEN - Share of individuals receiving social benefits,
UNEMPLOY - Unemployment rate, ELECTION - Election turnout, RSALARY - Average salary ratio, MANUALW - Share of manual workers.
