# Supplementary figures and images for: Individual- and area-level characteristics associated with alcohol-related mortality among adult Lithuanian males: A multilevel analysis based on census-linked data
Source: PLoS One. 2017 Jul 21;12(7):e0181622. doi: 10.1371/journal.pone.0181622 (PMC5521820; doi:10.1371/journal.pone.0181622)

**S2 Appendix**

**Administrative division of Lithuania**


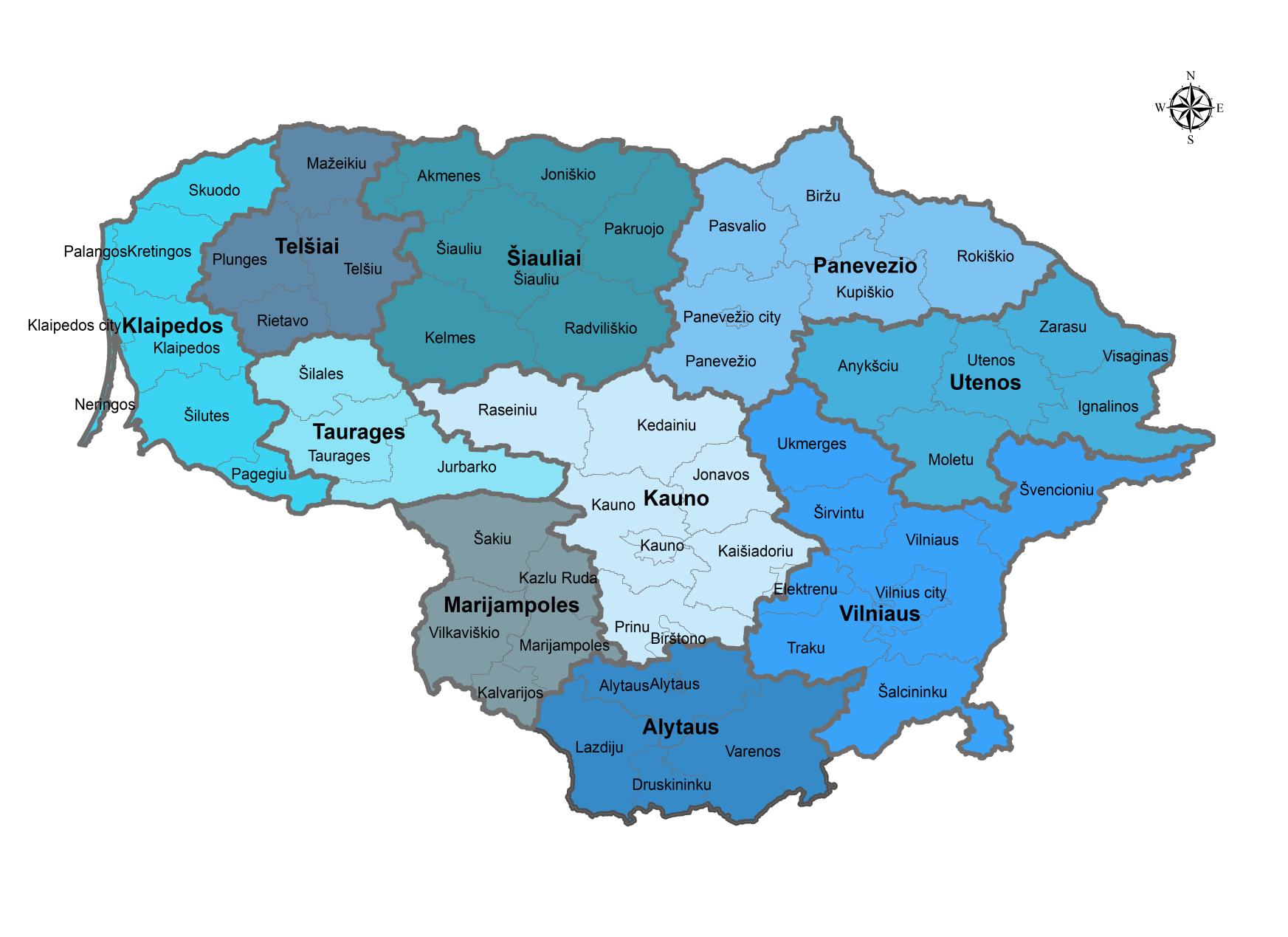

Supplement: S2 Appendix — (DOCX) [file pone.0181622.s002.docx]
